# Facilitators and barriers to co‐research by people with dementia and academic researchers: Findings from a qualitative study

**DOI:** 10.1111/hex.12891

**Published:** 2019-04-22

**Authors:** Jacob Waite, Fiona Poland, Georgina Charlesworth

**Affiliations:** ^1^ Research Department of Clinical, Educational and Health Psychology University College London London UK; ^2^ Greenwich CMHT for Older Adults, Memorial Hospital Oxleas NHS Foundation Trust London UK; ^3^ School of Health Sciences, Faculty of Medicine and Health Sciences University of East Anglia, Norwich Research Park Norwich UK

**Keywords:** barriers, co‐research, dementia, facilitators, peer research, qualitative

## Abstract

**Background:**

Public and patient involvement (PPI) is now established in dementia research. Barriers and facilitators to engagement from family carers and people in early stages of dementia have been explored. However, specific barriers and facilitators to co‐research with people with dementia have not previously been investigated.

**Objective:**

To discover the facilitators of, and barriers to, involving people with dementia as co‐researchers, from the perspectives of people with dementia, gatekeepers (family caregivers, ethics committee members, service providers) and researchers.

**Design:**

Thematic analysis of data from individual interviews about the co‐research experience.

**Results:**

Four themes emerged from interviews with 19 participants (five people with dementia): “getting one's head round it” (assumptions about research and dementia; different forms of language); practicalities (eg transport; accessibility of communication); “this feeling of safety” (perceptions of danger, protectiveness and opportunities for building trust); and motivations (“making a difference” and “keeping doing”).

**Conclusions:**

Findings both replicate and extend previous knowledge on PPI in dementia. Cognitive capacity of potential co‐researchers with dementia is only a part of the picture, with attitudes and expectations of researchers, gatekeepers and people with dementia also forming barriers. Researcher education, adequate resourcing, and both creativity and flexibility are needed to support recruitment of co‐researchers with dementia and to enable meaningful co‐research.

## 1 INTRODUCTION

In the UK, government health‐care policy stipulates “partnership between patients and clinicians in research,”^1^ and applicants to national funding bodies must describe public involvement.^2^ Service user involvement has taken the form of consultation and collaboration,^2^, ^3^ but now progressed to include co‐research where individuals do not merely comment on aspects of research but are actively involved in shaping the process.^4^, ^5^, ^6^ It is suggested that co‐researchers who have lived experience of the condition under investigation (“peer researchers”) can enhance the research process by, for example, identifying issues that may be overlooked by academic researchers^2^ or putting interviewees at ease by bring research closer to everyday life‐experiences and so enabling more open talk.^7^, ^8^ “Peer” research has been undertaken in populations that are unrepresented in researcher teams, for example, young people,^9^, ^10^, ^11^ people with intellectual disability,[12, 13, 14, 15, 16, 17] significant mental health difficulties^13^, ^17^, ^18^, ^19^ or people in later life.^13^, ^20^, ^21^ However, there have been few attempts to involve people with dementia as co‐researchers.

Until the 1990s, the perspectives and subjective experiences of people with dementia were largely absent from research.^22^ People with dementia have, by definition, significant impairments in two or more cognitive domains, such as memory, attention, perception, language and executive function, which necessarily impacts on an individual's ability to participate in, or engage with, research. Yet today the voice of the person with dementia is heard increasingly, in personal testimony, public consultation, service development and research.^23^, ^24^ Core principles for involving people with dementia in research have been co‐created,^27^ and barriers and enablers to the engagement of people with dementia in research have been identified.^28^ Of the 54 studies eligible for inclusion in a recent scoping review of engagement in dementia research, three studies involved people with dementia in data collection and eight described involvement in analysis.^28^ However, findings on barriers and facilitators to research engagement were derived from reflections of study authors rather than direct interview with (co‐)researchers.

The aim of the current study was to explore facilitators and barriers to people with dementia becoming co‐ or peer researchers, from the perspectives of people with dementia, “gatekeepers” to research and researchers.

## 2 METHODS

### 2.1 Approach

A “subtle realist” approach was adopted on the premise that we have multiple accounts, all of which are themselves shaped by various contexts, assumptions and beliefs.^29^, ^30^ Subtle realism encourages the use of accounts both as evidence about the phenomena they describe and as social constructions, reflecting beliefs and assumptions.

### 2.2 Participants

Participants were eligible if they (a) had direct or indirect experience of a research project that attempted (successfully or otherwise) to involve people with dementia as co‐researchers. (b) English language is fluent enough to take part in an interview (c) capacity to give informed consent.

### 2.3 Sampling Strategy and settings

Participants were purposively sampled from three groups: (a) researchers with experience of recruiting (or attempting to recruit) and working with people with dementia as peer researchers or co‐researchers; (b) “gatekeepers” to research, such as health, social care or voluntary sector staff, or family members who “stood between” a person with dementia and their potential involvement as a co‐researcher; and, (c) people with dementia with experience of co‐working with researchers. Recruitment took place through research team contacts, UK‐based researchers currently engaged in, or with published accounts of, attempts to recruit people with dementia as co‐researchers. People with dementia were recruited from voluntary sector organizations in the UK, and from among co‐researchers recruited to the PRIDE study. Gatekeepers were recruited via “snowballing” where one participant plays a part in recruiting subsequent participants. For example, participant researchers were asked whether they could name the ethics committee which had scrutinized their project(s). If they were able and willing to name the committee(s), then the committee administrator was contacted by JW and asked to circulate to the committee the information sheet for this interview study. Interested ethics committee members then made direct contact with JW .

Where sampling selections were made, this was done to maximize the breadth of the sample, with a minimum of five individuals per group.

### 2.4 Ethical approval

Ethical Approval for the recruitment of researcher and gatekeeper groups was obtained from the UCL Clinical Educational and Health Psychology Research Department's Ethics Chair (Ref: CEHP_2015_529) and from UCL Research Ethics Committee (Ref: 8635/011) for recruitment of people with dementia. Written informed consent was obtained from all participants using materials developed in conjunction with a public and patient involvement (PPI) group aligned to the PRIDE research programme.

### 2.5 Interview procedure

Interviews were carried out at a location of the participant's choice (eg home or workplace) and lasted around an hour. The topic guide for the interview was based on the Capability, Opportunity, Motivation model of behaviour change (COM‐B)^31^ with questions designed to elicit participants' views about the capabilities, opportunities and motivations that might enable or prevent a person with dementia undertaking co‐research. The term “capability” encompasses both physical and psychological (eg being able to engage in the thought processes necessary for the target behaviour, having capacity etc…); “opportunity” could be linked to the physical environment (eg being provided with transport) or the cultural environment (eg not invited to participate because of assumptions about abilities). “Motivation” includes processes that are intentional (eg “I want to make a difference”) or automatic (ie impulses and emotions arising from associated learning or innate dispositions).

The theory‐based questions were refined through discussion with the PRIDE PPI group. Then, after four interviews, minor revisions were made to allow more flexible use with researchers who had tried and failed to recruit people with dementia as co‐researchers and were therefore responding hypothetically. The study interviewer (JW) received advice on interviewing people with dementia from an Alzheimer's Society service‐used review panel.

### 2.6 Analysis

Interviews were transcribed verbatim, ensuring that any punctuation clarified the meaning of the original utterance. The five phases of thematic analysis were carried out by [initials removed for blind review].^32^ Initial codes were grouped, looking first for themes within each participant group, then looking across the whole data set. Themes and subthemes were refined through discussion with [names removed for blind review] and finalized in discussion with all authors.

## 3 RESULTS

### 3.1 Participants

Nineteen interviews were conducted: six academic researchers, eight gatekeepers and five people with dementia (Table 1).

**Table 1:** Participant characteristics

Participant no.	Age	Gender	Ethnicity	Group	Key relevant experience^a^
1	60	F	White	R	Attempted to recruit co‐researchers to do interviews
2	30	M	White	GK‐DC	Supported attempt to recruit co‐researchers
3	59	F	White	R	Carried out co‐research involving analysis
4	53	F	White	R	Attempted to recruit co‐researchers to do interviews
5	56	F	White	R	Carried out co‐research involving interviews and analysis
6	57	F	White	GK‐DC	Supported attempt to recruit co‐researchers
7	‐	F	Non‐White	R	Part of team, attempting to recruit interviewing co‐researchers
8	56	F	White	GK‐EC	Member of ethics committee which considered co‐research proposal
9	39	F	White	R	Carried out co‐research involving analysis
10	71	F	White	GK‐FC	Recruited as carer co‐researcher
11	–	M	Non‐White	GK‐FC	Carer co‐researcher
12	74	M	White	GK EC	Member of ethics committee which considered co‐research proposal
13	72	F	White	GK‐CC	Carer co‐researcher
14	56	M	White	PwD	Recruited as co‐researcher
15	59	F	White	GK‐CC	Carer to PwD recruited as co‐researcher
16	62	M	White	PwD	Experience of service user involvement (and as research participant)
17	73	F	White	PwD	Experience as interviewer of people with dementia for service evaluation
18	58	F	White	PwD	Experience as interviewer of people with dementia for service evaluation
19	–	M	White	PwD	Experience as interviewer of people with dementia for service evaluation

Note

Abbreviations: CC, current carer; DC, dementia charity employee; EC, ethics committee member; FC, former carer; GK, gatekeeper; PwD, person with dementia; R, researcher.

a Where an individual is described as having been recruited or attempting recruitment, this indicates their experience of co‐research does not go beyond this. “Carer co‐researchers,” here, are carers of people with dementia who interview other carers.

### 3.2 Themes

Four themes were extracted, each with subthemes:

*Theme 1: “getting your head round it”* refers to attitudes about feasibility of co‐research with people with dementia. Some participants were considerably more doubtful than others, with doubts themselves posing a barrier to recruitment.

*Subtheme 1: “fixed ideas” about research and dementia* influenced researchers' attitudes towards the co‐research enterprise. For example, one researcher described how they had been reluctant to recruit people with dementia as co‐researchers:

I had this sort of fixed idea of what dementia was [ ]. I thought people wouldn't be able to be involved in my research, that they wouldn't even consider it. (R1)

Another researcher described the incredulity of an ethics committee member:

“the world has gone mad. People with dementia, interviewing people with dementia. The world has gone mad” [ ] they just couldn't get their heads around it. (R5)

The researcher attributed this to a stereotyped view of the abilities of people with dementia:

I think they just thought “what is the point?” That they won't be able to understand what is going on, they won't be able to follow the conversation. [ ]” (R5)

Similarly, gatekeepers suggested that common assumptions about research (academic, complex, difficult) put people with dementia and carers off:

The barriers are there before you've even got to [explain the process], in terms of the word “research” and the thought “academic,” and the thought “complicated”. (GK6)

“Analysis”—no! Because that conjures up poring over and getting involved in detail. It would put me off for him. (GK15)

Some gatekeepers' assumptions about required research skills added to difficulties they foresaw. For example:

If you had dementia, would you remember enough of what was said to be able to lead **seamlessly** [*emphasis added*] into the next question? (GK10)

The speaker here expresses the idea of research as something technical, requiring skills unattainable for someone with dementia. This theme suggests that categorical definitions of dementia and research may be mutually contradictory when simultaneously applied to the term “co‐researcher with dementia.”

*Subtheme 2: the language of stages.* Contrasting with ideas that made it difficult for people to “get their head around it” were ideas that made it easier. When gatekeepers considered dementia as a series of stages rather than a homogenous category, it was easier to countenance the idea of a co‐researcher with dementia. For example, a carer, with previous personal experience of co‐research, expressed scepticism about the idea of people with dementia *in general* doing research:

I don't want to discredit any research but it's research isn't it? [ ] I don't know how they'd do it [ ] to me the inability to process could be a big stumbling block. (GK13)

But when she recalled a co‐researcher with dementia whom she had worked with, she explained this as an exception with reference to the “early stage”:

‐ it must have been early stage and she was probably alright. As I say she would sometimes forget at workshops but she would get through and it was fine. (GK13)

However, talking of stages inevitably draws attention to progression into a late stage, where the person is again seen as completely incapable (“losing it altogether” in the words of participant 12). Talk of doing research during the early stage is therefore often accompanied by concern about deterioration:

Who judges where the threshold is, the line in the sand is crossed, you know? (GK12)

The language of stages may enable envisaging someone with “early” stage dementia as co‐researching, while nonetheless raising the spectre of the “late” stage.

*Subtheme 3: noticing individual differences.* Also facilitating the idea of dementia co‐research was talk about individual differences among people with dementia. While one participant with dementia mentioned “stages,” others stressed diversity within the diagnosis:

Dementia is a thing of humans and humans are individuals and we are all different. (PwD17)

Researchers attributed stereotyping the capabilities of people with dementia to having only limited exposure to people with a dementia diagnosis. Those gatekeepers who endorsed the idea of co‐research often either drew on their own experience of people with dementia or pointed to culturally available images who were self‐evidently able. For example, one ethics committee member, after describing a colleague dismissing the idea of a person with dementia being involved in research, commented:

I mean (laughs) it was quite strange because at the same time you could turn on Radio 4 and hear [ ] Terry Pratchett articulating quite clearly what it was like to live with dementia. (GK8)

The talk of all six participant researchers reflected how co‐research was made easier to consider to contemplate when the individual service user perspective was seen to have intrinsic validity.

All three co‐research‐experienced researchers also reflected on the need to “learn from experience,” and let go of assumptions:

I learnt that if, really, you're serious about involving service users, you've got to be prepared to go where it takes you rather than staying on your fixed track. (R5)

*Theme 2: practicalities* refer to talk about cognitive demands, accessibility and resources.

*Subtheme 1: “good fit”* refers to talk about whether a person with dementia needs to have pre‐specified research skills, or whether the research can be tailored to the person's abilities. The label “good fit” is used to convey that any barriers would be identified and addressed by aligning the selection and design of research tasks to what people can do. Different people therefore emphasized different features of research tasks and appropriate abilities. People with dementia placed more emphasis on the *task* being a barrier. Imagining how analysis might work, one participant said:

I could sit and discuss what people had said with you, maybe helping you to understand but if you gave me rows of figures to analyse or the text, forget it! (PwD16)

Similarly, describing the interview task another participant remembered:

I asked the questions and [a supporter] scribed for me but I couldn't have done both, no way. (PwD19)

Researchers with co‐research experience laid more emphasis on tailoring the research activity to fit the abilities of the people with dementia. For a project involving both carers and people with dementia as co‐researchers, R5 described separating the two groups for data discussions:

…we thought [that otherwise] they won't have the space in the same way because other people will talk and things will move along too quickly. (R5)

Researchers without peer research experience placed more emphasis on finding people who “fit” the demands of the research tasks, so also foregrounding disabilities of the person with dementia. For example, in a project that had attempted to recruit people with dementia but had only involved carers, the researcher wondered whether people with dementia would have been able to “cope” with the analysis of full transcripts, as the carers had:

…there were a lot of people talking. A lot of issues were getting raised, a lot of stuff was getting written on flip charts [ ] I'm just wondering how they would cope. (R1)

A lack of relevant knowledge was also identified as a barrier:

One of the challenges we found was that there wasn't really guidance in how to do [co‐research with people with dementia]. (R9)

*Subtheme 2: accessibility*. Interviewees from all three participant groups raised issues of access, from hearing about the co‐researcher role in the first place to travelling to the relevant venues. The person with dementia is often dependent on others to pass on information:

…[he] would not get involved in things at all if I did not put things under his nose. (GK15)

I wouldn't go looking for the research because I didn't know it was out there but [my wife] knows .. you know she can use a computer better than I can. (PwD14)

Participants from all three groups suggested a face‐to‐face approach to recruitment was preferable:

He was saying about the method of recruitment [… ] he doesn't like doing stuff over the phone because he finds that hard to follow a conversation, and he struggles with the written word now. (GK6)

However, there are resourcing issues raised by face‐to‐face work:

Now, I could if I had the time to go into every single dementia café in the county [to recruit people with dementia face‐to‐face] but that was not my sole role. (R4)

Travel was referred to as an issue by most participants.

Somebody asks me would I like to do something, the first thing I think is “How am I going to get there”! (PwD18)

If the interviews were all over the place and [my husband] needed to get there [ ] then I have to get involved and ferry him all over the place. That gets difficult. (GK15)

*Subtheme 3: time constraints.* Addressing accessibility issues takes time and resources from the research team, but time constraints can also be an issue for co‐researchers. Participants from all three participant groups reflected that those people with dementia interested in co‐research were also likely be busy with other things:

I get the invites, I look at them, and I decide yes or no. In most cases, I'm already booked for something else. (PwD19)

The sense of limited time is often particularly acute for people with dementia, and participants from all three groups reflected that this might lead to a reluctance to commit to long‐term projects:

…research takes a long time, doesn't it? And I think sometimes we need to do it quicker because we don't know how much time we've got and you have to be aware of that. (PwD18)

*Theme 3: “this safe feeling”* refers to talk about building trust and a sense of safety in order to overcome perceptions of danger. Participants across all three groups spoke to this theme.

*Subtheme 1: fears of research and dementia.* All five participants with dementia saw research participation as desirable. However, four of the five shared negative perceptions of research, based, in two cases, on experiences of not receiving feedback after research participation:

…you never heard another word. It could be that my input was absolutely rubbish. I would still like to know because I thought “well, I won't do that again.” (PwD19)

Two talked about experiences of getting it “wrong” in front of “experts”:

…you've managed to get the confidence up to get involved with something like this [ ] and you are surrounded by all these experts who all know best anyway, and then they disagree totally with what you've said [ ]. Would you want to do it again? (PwD16)

Two interviewees spoke about fears around whether people with dementia might become overemotional, as well as whether they themselves could handle the emotional impact of interviews:

We were all a wee bit wary of visiting the care home, thinking are we going to upset these people, you know? And we knew it could possibly upset us (PwD18)

Among the gatekeeper group, the most frequently mentioned danger was that of emotional harm. This was most strongly articulated by two individuals (one a former a carer, the other a carer of a person with advanced dementia) who wondered whether this danger was so great that people with dementia should not do co‐research at all:

I really do think that there's a chance for someone doing the interviewing to be messed up where perhaps they were doing not too badly. (GK10)

There was a strong desire, particularly amongst former carers, to protect the people with dementia from perceived dangers:

If they were vulnerable, I think you would probably protect them rather then send them out there. (GK10)

From the perspective of researchers trying to recruit, and sometimes the person with dementia, the desire to protect was sometimes a barrier:

[Carers would say] “it will be upsetting for her, it will be too much. I'd rather you didn't carry on talking to her” (R5)

If [my wife] thought that something might upset me, she would put her foot down. And she's got a very big foot! (PwD19)

*Subtheme 2: comfort with self and others*. In contrast, participants from each group reflected that co‐research was facilitated if the person with dementia was at ease with both themselves and their diagnosis, and with the academic researcher with whom they worked. Gatekeepers and people with dementia made a comparison between people with dementia who are so distressed by their diagnosis that they prefer to isolate themselves and those who had “come to terms” with their diagnosis. Gatekeepers and people with dementia suggested the need for resilience in people with dementia to be able to interview others with the same condition and not be negatively affected by it:

…that's a very important thing, that you're able to look at people a lot worse than yourself and be able to go home and cope with it. (PwD19)

You've got to be comfortable in your own skin to be able to go and talk to somebody else and if you're not comfortable with it I think that would be very difficult. (GK10)

Similarly, participants from all three groups, but especially people with dementia, spoke about the importance of trust between co‐researcher and academic, particularly in relation to the interview situation:

I always had this safe feeling with her that if I got stuck I could just turn and ask her. Feeling safe is so important. (PwD19)

Those researchers and people with dementia who had engaged in peer research tended to describe the researcher as an enabling, supportive presence. There is a tension between these accounts and those of others who have not engaged directly in co‐research that imagine the researcher keeping the person with dementia safe in a different way, not so much supporting, as monitoring:

Whoever was supervising you would have to be watching you very closely because you, as a person with dementia, won't realise that you are deteriorating (GK10)

One person with dementia imagined the presence of the researcher not as reassuring but restrictive:

We should be left alone, not being controlled, there is a lot too much control, I feel, but that is my opinion. (PwD17)

These last excerpts perhaps highlight tensions within the researcher's role—trying both to protect and empower.

*Subtheme 3: familiarity.*All three participant groups identified factors that help create the necessary feeling of safety and security. These factors are collectively labelled “familiarity.” Doing the research activity somewhere familiar to the person with dementia is something that the co‐research experienced researchers said was helpful in making the activity feel comfortable. Already knowing the researcher was identified as important by people with dementia. Developing a relationship by creating opportunities for relaxed, unpressured talk—often over “cups of tea”—between researcher and co‐researcher was frequently described as helpful in developing a feeling of familiarity and trust:

You have to find a way of spending time, non‐productive time with the person, maybe a cup of coffee, a chat, where there's no pressure on anything that is going on, to allow a relationship to initiate. (PwD16)

The speaker's plea here for time to allow relationship building is echoed by a researcher remembering their decision not to prioritize efficiency when deciding to drive co‐researchers to and from interviews:

I could have easily thought “Oh let's buy taxis" to save me, you know, driving around but actually that whole bit of picking them up and having a chat and driving them home and having a chat, all of that I think was quite important (R5)

Overall, the talk within this subtheme suggested a degree of consensus across the different groups of the value of familiarity and sustained relationships.

*Theme 4: motivations* theme refers to talk about reasons why participants from different groups might, or might not, actively want a person with dementia to engage in co‐research.

*Subtheme 1: Making a difference.* Across all three participants groups, participants spoke about people with dementia participating in co‐research out of desire to “make a difference,” especially for other, future, people with dementia:

He has a very firm view that he wants to do everything he can to improve the situation for the generations to follow. (GK15)

Another aspect of “making a difference” was the experience of one's words and actions having a tangible effect. Two participants with dementia had carried out interviews as a part of an evaluation of care homes. One remembered how their opinion was decisive in determining whether they should inform staff of their diagnosis:

…and I said “Yes we do,” that's it! “Because you have no idea what I would like if I was in a care home.” So that's what we did (proudly). (PwD18)

The second remembered how they had pointed out some uneven carpet as a potential hazard:

…so I said “That lady won't see that!” to the manager. “And it needs to be flattened,” so before we left it was flattened. (PwD19)

The detail with which people with dementia described discrete instances of “making a difference” contrasted with the more generalized way they talked about the more common experience of “tokenism” (where a person with dementia is invited to attend a meeting simply so that the claim can be made that they were involved):

We weren't given the opportunity to speak, we weren't included in anything, we were just there, so they could say they “had” you. (PwD14)

Participants from all three groups refer to the dangers of tokenism. While the emphasis for the people with dementia is on the experience of invalidation, the researchers' focus is on resisting the urge to recruit people just to fulfil the research brief:

We wouldn't have wanted someone with dementia just sitting there just for the sake of saying oh we've got someone with dementia involved (R5)

In terms of what enables “making a difference,” there are some tensions within the data. Two of the researchers talk about participants contributing as much or as little as they want, to accommodate those whose ability to be involved is limited:

You know it's as much time as you feel you can give. We're also interested in your views on our analysis. So it's as much time as you can give. (R4)

Arguably, though, by locating this limitation in ability to be involved inside the people with dementia, these researchers are avoiding the question of whether they might not create, for example, a shorter project which would enable the person to participate more fully. One person with dementia saw this kind of attempt at inclusion as more tokenism:

If your involvement is that haphazard, are you actually involved in it? Or are you just going along and saying “Oh, we've got so and so and they've been diagnosed with…as part of our team.” (PwD16)

There are further tensions regarding the differences that *researchers* hope to make through co‐research. While most researchers saw co‐research as potentially empowering people with dementia, there was more ambivalence as to whether it would make a positive difference to research data. Those who had carried out co‐research saw a value in the additional perspective brought by the person with dementia; others were more ambivalent:

What were we doing it for? Were we expecting it to make a difference to the data? (R4)

*Subtheme 2: “Keeping doing”* refers less to having an effect on others or the immediate environment and more to remaining engaged in life, sometimes with an idea of holding dementia at bay, sometimes of maintaining one's pre‐diagnosis identity:

It just fed into her own personal outlook and past history of being someone who was very inquisitive. (R3)

…something that takes him out of the home and engaging with other people [ ] it gives him something else to think about. (GK15)

It makes me use my brain. Doing different things keeps you doing, you know? (PwD17)

In the main, participants saw this “keeping doing” as a positive reason for engaging in co‐research. However, there was one exception; one participant within the gatekeeper group described how for some people, for example those who are retired and see a positive value in no longer being at work, the thought of being a researcher is quite unattractive:

As far as they're concerned, they've done their job, this is a job, being in research is a bit like a job, and if you're old and you've retired, I don't want to go and sit and talk to an academic, I really don't. (GK6)

So, with one exception, the “keeping doing” subtheme represents a means of the person with dementia staying engaged with life, or maintaining valued aspects of their identity.

## 4 DISCUSSION

Findings from this interview‐based, qualitative study on the involvement of people with dementia in co‐research both replicate and extend knowledge of facilitators and barriers to PPI engagement. Comparing findings from this study with Bethell's recent review,^28^ barriers in common include the following: time and costs; “gatekeeper” attitudes; difficulty identifying “representative” individuals and groups; (actual or perceived) complexity the research process; lack of training and experience; and the potential for distress.^28^ Similarly, facilitators in common include the following: early planning by researchers, including clarity of role definition, careful consideration of consent and capacity, and practical planning for dementia‐friendly meetings (eg familiar surroundings, regular breaks, help with travel); having appropriate and adequate resources (eg time, training and funding); good relationships between co‐researchers and institutions, including ethics boards, funding agencies, health charities and volunteer groups; ensuring clear and jargon‐free communication that is supportive of people with dementia including regular updates on study progress, results and outcomes, and acknowledging contributions; and maintaining flexible attitudes and approaches, taking into account each person's individual strengths, skills, preferences and needs, and acknowledging that, with dementia, circumstances can change over time.^28^

Findings from the current study emphasize the importance of attitudes not only towards people with dementia, but the *combination* of people with dementia and research. A parallel can be drawn with the intellectual disabilities literature, where clinicians emphasize cognitive barriers,^33^, ^34^ whereas people with intellectual disabilities lay greater emphasis on the research as a barrier.^33^ It takes time for researchers who are new to the field of co‐research or dementia to see people with dementia as individuals with knowledge and experience rather than members of a category associated only with impairment. Similarly, people with dementia and their families who do not have a history of conducting research may not immediately consider the possibilities of engaging with a co‐research role. Co‐research is facilitated by a “good fit” between the research tasks and the co‐researchers' aptitudes and abilities. “Fitting” may involve adapting the task to the person^35^ or finding the person for the task. Creativity, flexibility and careful planning are required to achieve a balance between scientific integrity and a “good fit” for co‐researchers, but key attributes for academic researchers appear to be the willingness to take time to engage and frequently re‐connect with co‐researchers.

The current study extends previous findings of the importance of good relationships and communication to highlight both ontological and interpersonal safety. The theme of feeling “safe” encompassed not only the relationship between researchers and collaborators with dementia but also the need for the person with dementia to be at ease with themselves and their diagnosis. The benefits of developing a sense of familiarity and trust have been emphasized previously for people with dementia engaged in social change.^36^

Finally, this study extends the knowledge of motivations of people with dementia to engage with co‐research opportunities. It is evident that the co‐research role is not something that is likely to be of interest, or within the capacity, of many people with dementia. A primary motivation for those that do engage is the person with dementia's desire to help others; a factor previously identified for carers of people with dementia in research and older co‐researchers both with and without dementia.^21^, ^37^ In contrast, the more latent aspect of deriving pleasure from *seeing the impact of your actions* was more specific to people with dementia and has not been identified previously in the co‐research context. The desire to “carry on engaging with life” was also an important motivation and is in keeping with the dementia literature around “valued identities” and the desire to maintain a sense of continuity with the pre‐diagnosis self.^28^, ^38^, ^39^

### 4.1 Strengths and limitations

The data collection for this study took place during a time of development in PPI, including a change in terminology used to describe the person with dementia working with an academic researcher from “peer researcher” to “co‐researcher”. We moved to adopt the new terminology as the term “co‐researcher” seems a better fit than “peer” for circumstances where there can be wide variation between a research participant with dementia and a researcher with dementia in terms of the specific dementia diagnosis and cognitive abilities. Since the completion of the project, European guidelines have been published on the involvement of people with dementia in PPI which includes the term “co‐researcher” but not “peer researcher.”^40^

Secondly, recruitment to this study was limited to voluntary and University sectors, and not NHS channels (aside from NHS ethics committee members). Although the original plan was to include NHS recruitment, the actual sample better represents the current practice of co‐research in the UK, given the governance arrangements for co‐research in NHS settings carry a high level of burden.^3^

Thirdly, there was a protocol change in that the original intention to use COM‐B as a coding framework for analysis was abandoned in favour of a “bottom‐up” analytic approach. During the analysis phase, it became apparent that coding within the COM‐B model would result in the separation of themes in a way that obscured participants' contributions. For example, the “good fit” subtheme encompassed the interplay between two categories that are distinct in COM‐B, namely individual capabilities and opportunities. Similarly, the “this safe feeling” theme comprised talk about feelings (fear, comfort) and conditions that gave rise to those feelings (having time to get to know the researcher) which, within the COM‐B model, would be categorized as motivations (ie emotions arising from associative learning) and opportunities, respectively.

Although sampling procedures were designed to maximize diversity, the majority of participants were white and female. All people with dementia had only “mild” impairments, and only one participant with dementia had direct experience as a co‐researcher on a designated “research” project; others had experience of involvement in service evaluations, with three people with dementia taking on the interviewer role. Saturation was achieved with the existing sample, but different themes may have emerged from a more diverse sample. Furthermore, there are some ambiguities in the data where it is not clear whether participants are referring to their experience as a co‐researcher or to their additional experience as a research participant. Potential methodological limitations are the inclusion of some interviewees being known to the supervisors of this project (with the risks associated with “insider research”) and the inclusion of three types of participant within the same qualitative analysis—researchers, gatekeepers and people with dementia. A further limitation is that all participants with dementia were interviewed towards the end of the interview series due to the time associated with the higher level of ethical scrutiny for the involvement of potentially vulnerable people compared to the procedure for “healthy adults.” However, despite differences between and within groups in terms of individual positioning, there were themes that ran through the entire data set.

### 4.2 Implications

#### 4.2.1 Academic researcher training

Before embarking on co‐research with people with dementia, academic researchers should not only familiarize themselves with available guidance,^27^, ^40^ but also examine their own assumptions about “research” and “dementia” to identify unhelpful stereotypes.

*Recruitment of co‐researchers with dementia* could be facilitated by ensuring that aversive language (eg decline, later stages) is not used in information sheets and that the potential benefits described are those that are meaningful to people with dementia (eg making a difference; building on existing skills). Direct recruitment (rather than via gatekeepers) may also be relevant, as has previously been advocated in the intellectual disabilities field.^41^

#### 4.2.2 Balancing the right to be involved with protection from potential harm

The newness of the co‐researcher role in dementia means that a balance has yet to be struck between protection of potentially vulnerable people and the right to be involved. It is vital that researchers have considered and addressed potential harms, from tokenism^40^ to overwhelming cognitive or emotional demands. Tokenistic involvement is to the detriment of the co‐researcher with dementia and brings research into disrepute. At the other end of the spectrum, involvement in activities that are too cognitively or emotionally intense will risk distress and thus reinforce the belief that the involvement of people with dementia as co‐researchers is harmful to well‐being. The fear of emotional consequences from exposing co‐researchers to people with more advanced dementia reflects wider societal fears of exposure to the fourth age.^42^ While there are indeed potential risks to involving co‐researchers with dementia, we can seek to avoid or manage such risks rather than veto the co‐research enterprise.

#### 4.2.3 Future research

Any future qualitative explorations of the co‐researcher experience may benefit from including non‐research‐active family carers who are gatekeepers for a co‐researcher with dementia (given the potential for differences in perspectives between carers who are, and are not, co‐researchers) and from providing alternatives to the “traditional sit down interview” for data gathering with people with dementia.

## 5 CONCLUSIONS

People with dementia engaging with the co‐research role often see it as important to continue to make a difference, gaining a sense of satisfaction from making a meaningful impact. The cognitive profile of people with dementia has practical implications for research, but this, and other barriers to participation, can be often be addressed with adequate time, resources, creativity and flexibility to find a “fit” between the person and the task. Methodologies from other contexts may help in achieving the involvement of co‐researchers with dementia.

## CONFLICT OF INTEREST

None.
